# Asymmetric Myocardial Involvement as an Early Indicator of Cardiac Dysfunction in Pediatric Dystrophinopathies: A Study on Cardiac Magnetic Resonance (CMR) Parametric Mappings

**DOI:** 10.1007/s00246-024-03488-8

**Published:** 2024-04-30

**Authors:** Roger Esmel-Vilomara, Lucía Riaza, Laura Costa-Comellas, Anna Sabaté-Rotés, Ferran Gran

**Affiliations:** 1 Faculty of Medicine, Universitat Autònoma de Barcelona, Barcelona, Spain; 2 Pediatric Cardiology, Vall d’Hebron Hospital Campus, Carrer Sant Quintí 89, Barcelona, 08041 Spain; 3 Pediatric Cardiology, Hospital de la Santa Creu i Sant Pau, Institut d’Investigació Biomèdica Sant Pau (IIB Sant Pau), Barcelona, Spain; 4 Pediatric Radiology, Vall d’Hebron Hospital Campus, Barcelona, Spain; 5 Pediatric Neurology, Vall d’Hebron Hospital Campus, Barcelona, Spain

**Keywords:** Dystrophinopathies, Cardiac magnetic resonance, Parametric mappings, Cardiomyopathy, Heart failure

## Abstract

Dystrophinopathies, such as Duchenne and Becker muscular dystrophy, frequently lead to cardiomyopathy, being its primary cause of mortality. Detecting cardiac dysfunction early is crucial, but current imaging methods lack insight into microstructural remodeling. This study aims to assess the potential of cardiac magnetic resonance (CMR) parametric mappings for early detection of myocardial involvement in dystrophinopathies and explores whether distinct involvement patterns may indicate impending dysfunction. In this prospective study, 23 dystrophinopathy patients underwent CMR with tissue mappings. To establish a basis for comparison, a control group of 173 subjects was analyzed. CMR protocols included SSFP, T2-weighted and T1-weighted sequences pre and post gadolinium, and tissue mappings for native T1 (nT1), extracellular volume (ECV), and T2 relaxation times. The difference between the left ventricular posterior wall and the interventricular septum was calculated to reveal asymmetric myocardial involvement. Significant differences in LV ejection fraction (LVEF), myocardial mass, and late gadolinium enhancement confirmed abnormalities in patients. Tissue mappings: nT1 (*p* < 0.001) and ECV (*p* = 0.002), but not T2, displayed substantial variations, suggesting sensitivity to myocardial involvement. Asymmetric myocardial involvement in nT1 (*p* = 0.01) and ECV (*p* = 0.012) between septal and LV posterior wall regions was significant. While higher mapping values didn’t correlate with dysfunction, asymmetric involvement in nT1 (ρ=-0.472, *p* = 0.023) and ECV (ρ=-0.460, *p* = 0.049) exhibited a significant negative correlation with LVEF. CMR mappings show promise in early myocardial damage detection in dystrophinopathies. Although mapping values may not directly correspond to dysfunction, the negative correlation between asymmetric involvement in nT1 and ECV with LVEF suggests their potential as early biomarkers. Larger, longitudinal studies are needed for a comprehensive understanding and improved risk stratification in dystrophinopathies.

## Introduction

Dystrophinopathies are X-linked recessive neuromuscular disorders stemming from pathogenic dystrophin gene variants (*DMD*, locus Xp21.2). They encompass two well-recognized conditions: Duchenne muscular dystrophy (DMD), with an incidence of 1 per 3.800–6.300 live births males, and the comparatively milder and less frequent Becker muscular dystrophy (BMD) [1–3]. In DMD, the absence of dystrophin, a subsarcolemmal protein, induces progressive muscle degeneration, leading to the loss of independent walking, respiratory insufficiency, and cardiomyopathy during adolescence or early adulthood. BMD is characterized by a reduction in dystrophin levels, and the onset of symptoms generally occurs later in life, with a slower progression [2, 4, 5]. In addition, around 10% of DMD female carriers exhibit certain clinical manifestations of the disease like mild weakness, isolated cardiomyopathy or cognitive involvement [1].

Cardiac dysfunction, which eventually progresses to dilated cardiomyopathy with heart failure, stands as one of the primary causes of mortality in these patients [1, 5, 6]. Detecting this condition is challenging and usually relies on imaging findings, as the manifestation of symptoms is obscured by limited patient mobility [2, 4, 6]. Hence, establishing proactive strategies for early diagnosis becomes crucial in enhancing the quality of life for those affected [5].

While there is no current cure for DMD, the use of glucocorticoids aims to extend ambulation, delay cardiomyopathy, and enhance life quality while slowing disease progression [2, 4]. In the last years, research focuses on developing novel therapies to restore dystrophin function, including exon skipping, stop codon readthrough, gene replacement and gene editing [7]. However, tools for early cardiac dysfunction assessment and treatment response evaluation are still in development. Traditional imaging methods, like echocardiography and cardiovascular magnetic resonance imaging (CMR), excel in assessing heart structure and function but lack insight into subclinical microstructural remodeling, identified on pathology as progressive fibrofatty infiltration [8–10]. Parametric CMR tissue mappings hold potential for evaluating microstructural changes, primarily fibrosis [11–13], and potentially detecting early heart involvement in this high-risk group [8–10, 14].

The objective of this study is to characterize tissue features (fibrosis, edema and fat) through CMR parametric mappings within a cohort of pediatric patients diagnosed with dystrophinopathies. Additionally, we seek to discern whether any pattern of myocardial involvement may work as a potential indicator for incipient cardiac dysfunction.

## Methods

This prospective study comprised pediatric patients affected by dystrophinopathies (*n* = 23), who underwent CMR with tissue mappings between May 2019 and September 2023. Patients with DMD were classified into four stages based on their motor abilities: pre-symptomatic (no symptoms, only creatine kinase elevation), early ambulatory (able to rise from the floor and climb stairs with some difficulty), late ambulatory (inability to climb stairs or rise from the floor), early non-ambulatory (wheelchair mobility with good trunk control), and late non-ambulatory (loss of upper limb and trunk control) [1, 5].

To establish a basis for comparison, we analyzed normal CMR data from subjects (*n* = 173) who underwent CMR for non-myocardial diseases such as heart block and ventricular ectopy and had no myocardial involvement.

Echocardiographic studies were conducted to evaluate the right and left ventricular (LV) ejection fraction (LVEF), hypertrophy, and dilation. LVEF was assessed using Simpson’s biplane method, with an LVEF < 50% indicating LV dysfunction. LV dilation was defined as an end-diastolic diameter z-score > + 2. Valvular regurgitation was evaluated by assessing the color flow area of the regurgitant jet and its extension into the atrium. Electrocardiograms (ECG) were utilized to identify specific abnormalities, including deep Q waves (> 2 mm) and T-wave inversions.

**Fig. 1:**
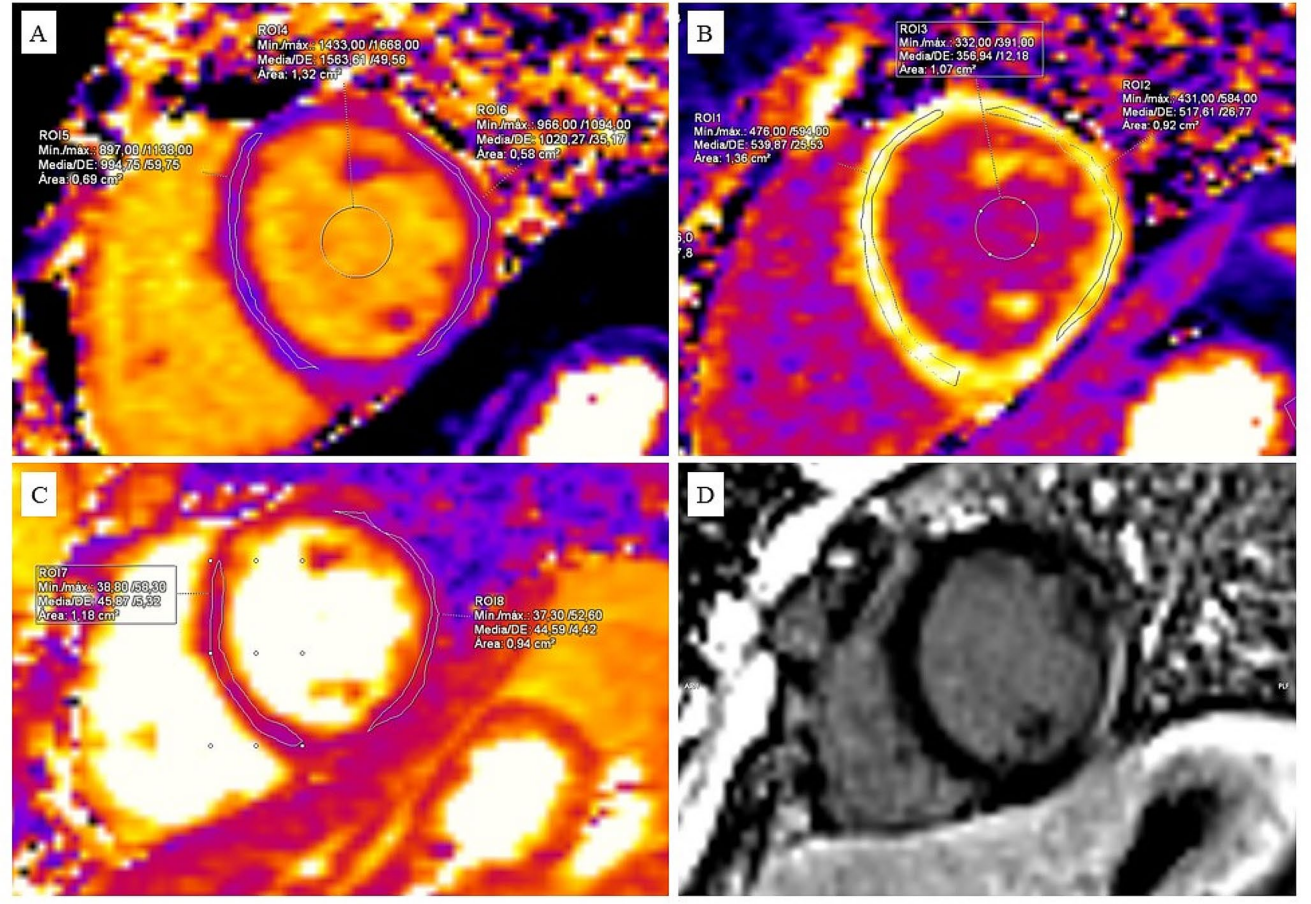
Examples of tissue mappings and late gadolinium enhancement (LGE) in a patient with Duchenne muscular dystrophy. T1 mappings (**A** and **B**) and T2 mappings (**C**) were measured in the LVPW and IVS during end-diastole in short-axis views. Native T1 mappings pre-contrast (**A**) and post-contrast (**B**) were used to calculate ECV. Image **D** shows late gadolinium enhancement in the LVPW

Cardiac MRI was conducted using a 1.5 T Magnetom Avanto (Siemens Medical System) with cardiac synchronization. Assessment of ventricular volumes, function, and myocardial mass was performed using balanced steady-state free precession sequences (SSFP), acquired as a short-axis stack. Dysfunction was identified by an ejection fraction below 50%. T2-weighted short-tau inversion recovery (T2W-STIR) and T1-weighted sequences (TSE) were performed before and after the administration of intravenous gadolinium. Phase sensitive inversion recovery (PSIR) reconstructions enabled the delayed uptake of contrast detection, commonly known as late gadolinium enhancement (LGE). Tissue mappings were measured on the left ventricular (LV) posterior wall (LVPW) and the interventricular septum (IVS) during end-diastole in short-axis views, and encompassed native T1 relaxation times (nT1), extracellular volume (ECV), and T2 relaxation times. For ECV, a modified look-locker inversion recovery sequence (MOLLI) on T1 was used, before and 20 min after contrast administration (Fig. 1). The difference between LVPW and IVS was calculated to reveal asymmetric myocardial involvement. Normative data for nT1, ECV and T2 is provided [15, 16].

Statistical analysis was carried out using SPSS 25.0 (IBM). Nominal data were described using proportions, and continuous quantitative data as medians and interquartile range [IQR] due to the non-normal distribution of the sample. The Mann-Whitney U test was employed to assess continuous variables, and Spearman’s Rank was used to determine correlations (ρ). P values < 0.05 were deemed statistically significant.

## Results

A total of 23 pediatric patients were included in the study, comprising 14 DMD cases, 1 female carrier, and 8 BMD cases. Among BMD patients, all were ambulatory, whereas within the DMD group, 5 were in the early ambulatory stage, 1 in the late ambulatory stage, and 8 in the late non-ambulatory stage. Demographic information is shown in Table 1.

Among boys with DMD, a reduced LV ejection fraction (LVEF) was detected in 3 out of 14 (21.4%) through echocardiography and in 4 out of 13 (28.6%) through CMR. Among BMD patients, a reduced LVEF was observed in 1 boy via echocardiogram and in 2 out of 8 (25%) through CMR. Metrics of LV function derived from echocardiography, standard CMR, and tissue mappings are presented in Table 1. As expected, significant differences were evident between patients and controls in LVEF (59 vs. 65%, *p* = 0.01), myocardial mass (66 vs. 54 g/m^2^, *p* = 0.038), and LGE (12/23 vs. 1/171, *p* < 0.001). Regarding tissue mappings, significant differences were observed in nT1 at the LVPW (1038 vs. 991 ms, *p* < 0.001), while no significant differences were noted at the nT1 in the IVS, nor in ECV or T2-weighted mappings.

**Table 1:** Description of echocardiographic and cardiac Magnetic Resonance characteristics, stratified by disease classification. Categorical data is presented as n (%) and continuous variables as medians [IQR], unless otherwise specified. Myocardial segments with LGE were classified into 5 areas based on the American Heart Association (AHA) 17-segment model [17]: anterior (segments 1, 7, 13), septal (2, 3, 8, 9, 14), inferior (4, 10, 15), lateral (5, 6, 11, 12, 16) and basal (17). Abbreviations: ACEi: angiotensin-converting enzyme inhibitors, MRI: Magnetic resonance imaging, LV: Left Ventricle, LVEF: LV Ejection Fraction, EDVI: end-diastolic volume index, ESVI: End-systolic volume index, IVS: Interventricular septum, LVPW: LV posterior wall, LGE: Late gadolinium enhancement, TDI: Tissue Doppler imaging

	Duchenne muscular dystrophy (*n* = 14)	Female carriers (*n* = 1)	Becker muscular dystrophy (*n* = 8)	Control group (*n* = 173)
**Age at evaluation**	13.5 [10.8–18.4]	17	16.5 [13.5–17.8]	13 [10.2–16.0]
**Medication**				
Steroids	14 (100%)	0 (0%)	3 (37.5%)	
ACEi	14 (100%)	1 (100%)	3 (37.5%)	
Beta-blockers	7 (50%)	0 (0%)	0 (0%)	
**ECG findings**	4 (28.6%)	0 (0%)	2 (25%)	
Deep Q waves	2 (14.3%)	0 (0%)	0 (0%)	
Negative T waves in				
lateral and inferior leads	2 (14.3%)	0 (0%)	2 (25%)	
**Echocardiography**				
Ejection fraction (%)	57.0 [53.8–64.5]	66.0	66.5 [60 − 0–72.0]	
LVEDV (z-score)	-0.4 [-1.5–0.1]	1.0	0.3 [-1.3–0.9]	
Mild mitral regurgitation	1 (7.1%)	0 (0%)	0 (0%)	
TDI s’ mitral wave (cm/s)	8.5 [8.0–12.0]	9.0	10.0 [7.0–12.8]	
**Cardiac MRI**				
LVEF (%)	58.0 [46.8–62]	61.0	60.0 [50.8–65.0]	65.0 [59.0–68.0]
LV EDVI (mL/m^2^)	64.0 [61.0 -76.3]	75.0	87.5 [66.8–99.5]	79.5 [70.0–89.8]
LV ESVI (mL/m^2^)	29.0 [22.3–34.0]	29.0	33.5 [28.0–42.0]	28.0 [24.0–34.0]
LV mass index (g/m^2^)	57.5 [49.75–72.5]	37.0	73.5 [66.0–83.0]	54.0 [47.0–65.0]
LGE	9 (64.3%)	1 (100%)	2 (25%)	1 (0.6%)
* Anterior	0/9 (0%)	0/1 (0%)	0/2 (0%)	0/1 (0%)
* Septal	1/9 (11.1%)	0/1 (0%)	2/2 (100%)	0/1 (0%)
* Inferior	1/9 (11.1%)	0/1 (0%)	1/2 (50%)	0/1 (0%)
* Lateral	9/9 (100%)	1/1 (100%)	2/2 (100%)	1/1 (100%)
* Basal	0/9 (0%)	0/1 (0%)	0/1 (0%)	0/1 (0%)
Native T1 mapping (ms)				
* IVS	1019.0 [986.0–1052.5]	942.0	987.5 [969.8–1019.0]	1006.0 [986.0–1030.0]
* LVPW	1043.0 [1032.5–1076.0]	975.0	1002.5 [987.5–1079.8]	991.0 [970.0–1018.0]
Extracellular volume (%)				
* IVS	24.1 [23.2–26.2]	27.6	24.5 [22.4–25.6]	26.7 [24.5–28.6]
* LVPW	26.4 [24.8–29.5]	27.4	24.2 [21.7–33.3]	25.0 [22.8–27.4]
T2 mapping (ms)				
* IVS	48.0 [46.0–49.5]	55.0	47.5 [44.0–46.3]	48.0 [45.0–52.0]
* LVPW	48.0 [45.0–51.0]	54.0	47.0 [44.0–46.0]	48.0 [45.0–51.0]

**Fig. 2:**
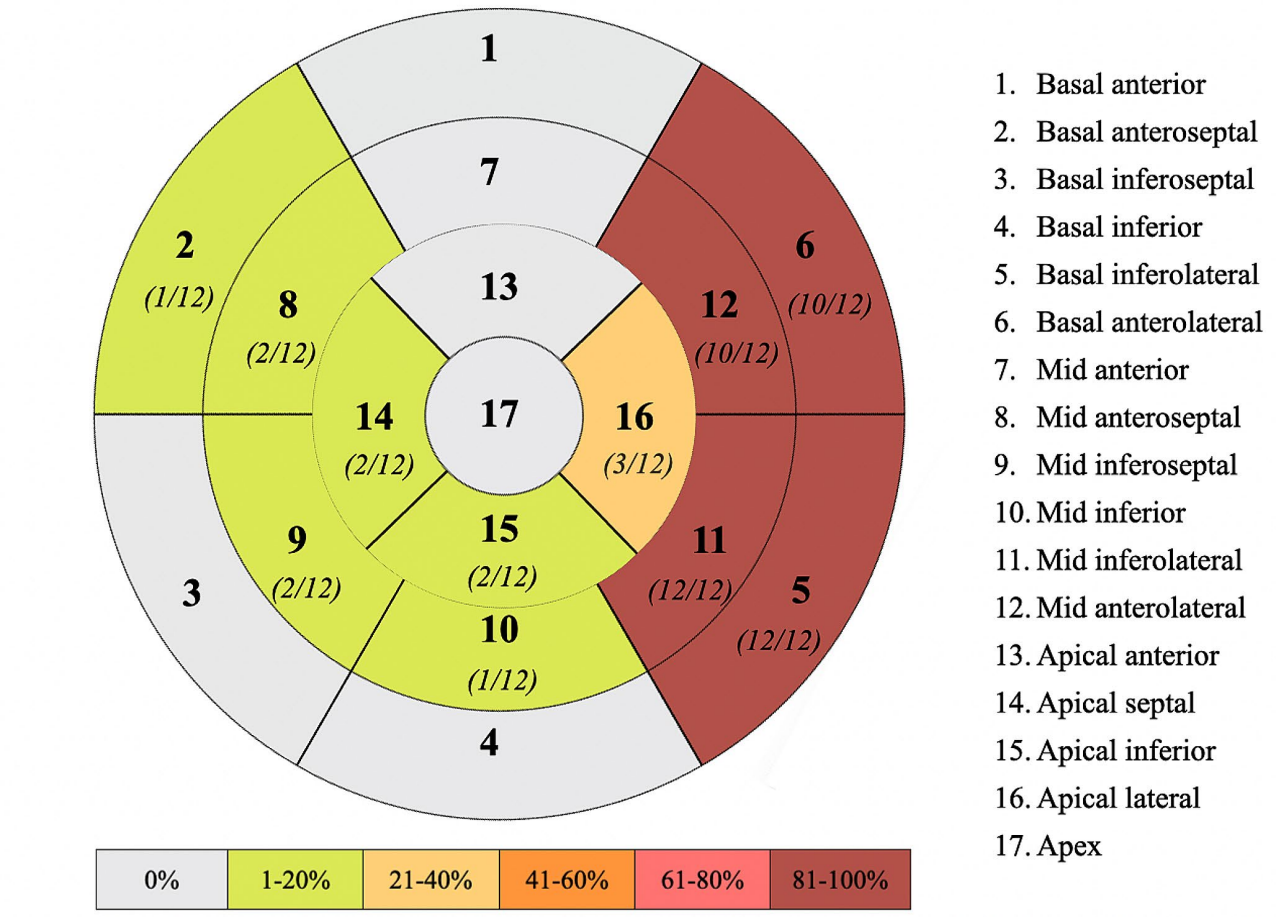
LGE affected segments. The majority of the affected segments are found in the lateral LV wall

Twelve patients demonstrated LGE in at least 4 myocardial segments based on the American Heart Association (AHA) 17-segment model [17], as illustrated in Fig. 2; all these patients presented involvement of the LVPW.

**Fig. 3:**
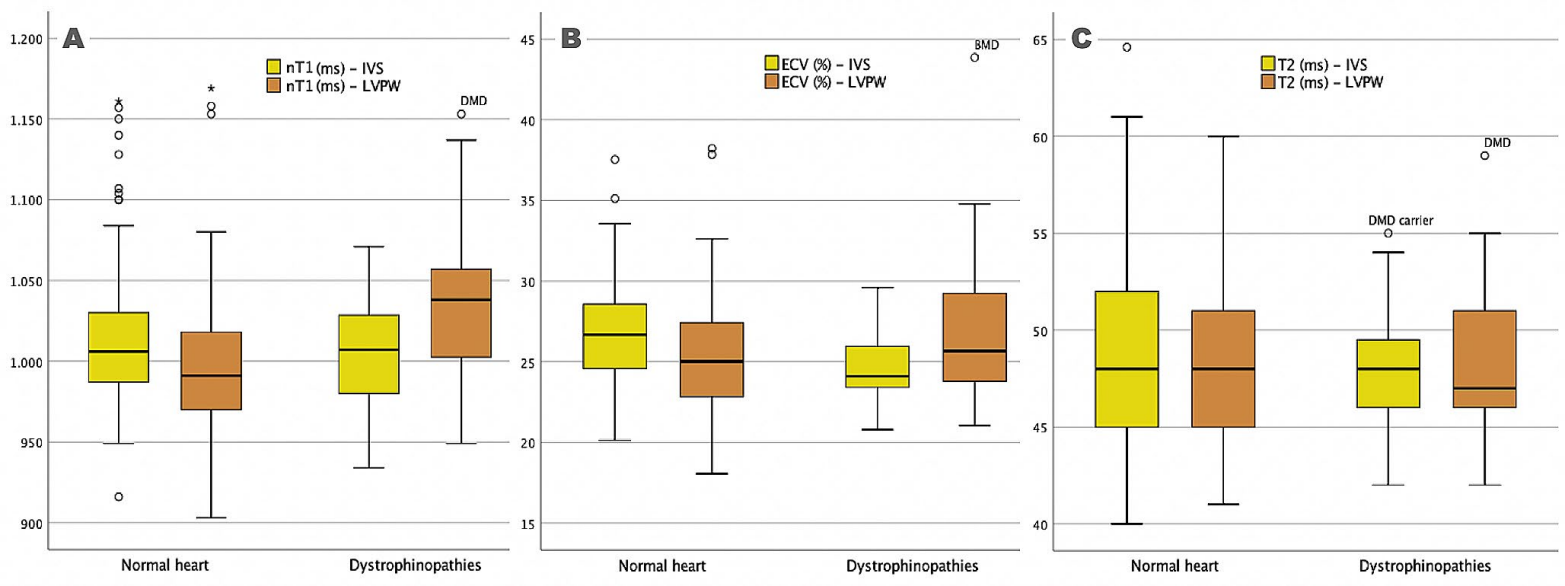
Boxplots illustrating the asymmetric distribution of tissue mapping values in the interventricular septum (IVS) and left ventricular posterior wall (LVPW) for **(A)** Native T1 (nT1), **(B)** Extracellular Volume (ECV), and **(C)** T2-weighted sequences. The asymmetry is presented in nT1 and ECV but not in T2-enhanced sequences

In patients with dystrophinopathies, an asymmetric myocardial involvement was detected, with statistically significant differences between septum and LVPW regions in nT1 (1038 vs. 1007ms, *p* = 0.01) and ECV (25.7 vs. 24.1%, *p* = 0.012), but not in T2 (48 vs. 47ms, *p* = 0.888), as represented in Fig. 3. This asymmetric involvement was distinctive of the patients as compared to controls in terms of nT1 (with a difference of 27 vs. 19 ms, *p* = 0.045) and ECV (with a difference of 2.9 vs. 1.5%, *p* = 0.019). In this small sample, a greater degree of asymmetry in the nT1 or ECV values wasn’t found to be associated with DMD in comparison to BMD, nor did it correlate with more advanced clinical stages in patients. Similarly, the presence of LGE in any segment was not associated with significant asymmetric involvement detected by CMR mappings.

**Fig. 4:**
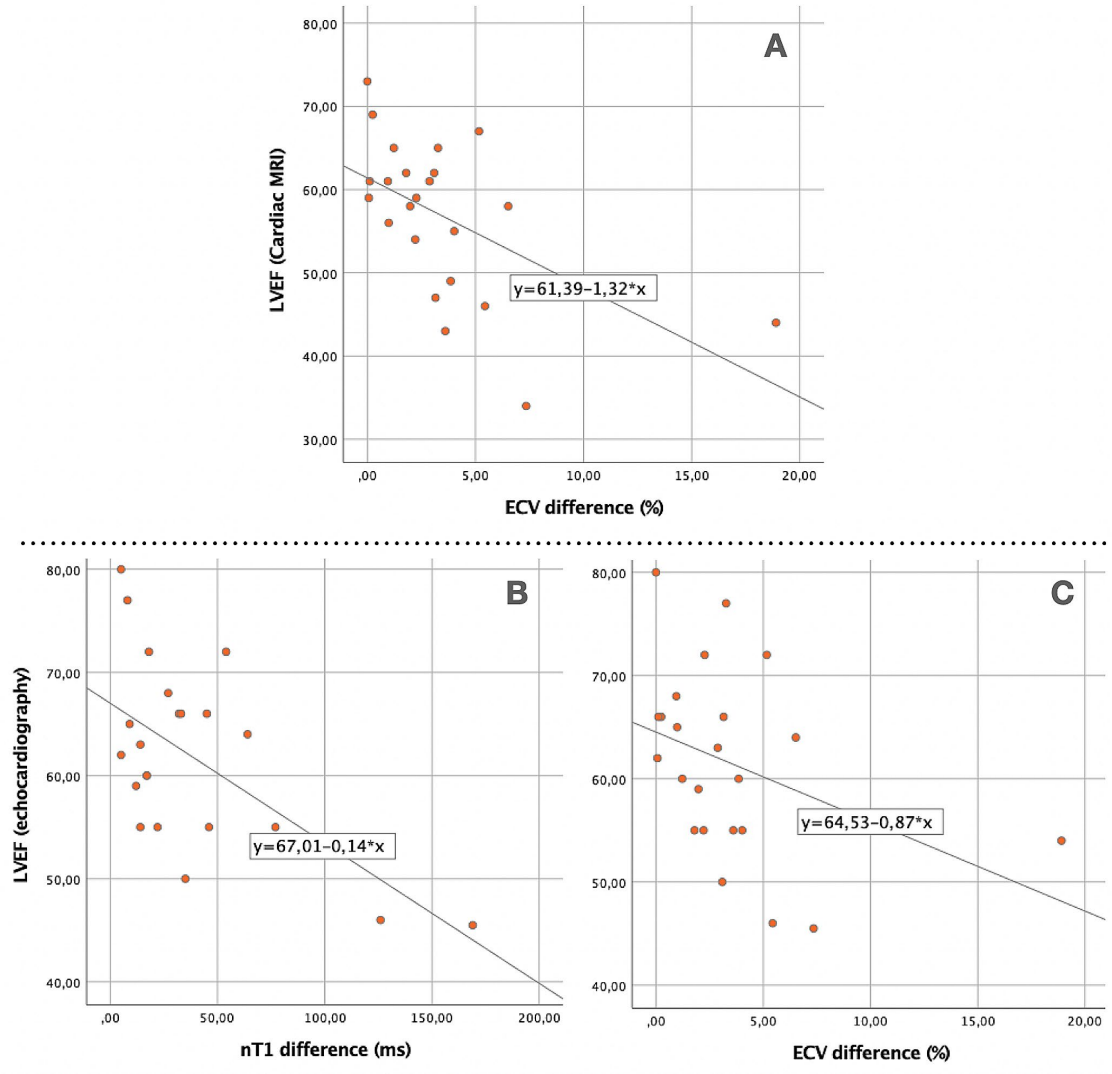
Scatterplots examining the correlation model between asymmetric myocardial involvement detected by ECV (**A** and **C**) and native T1 mapping (**B**) and cardiac function assessed by MRI (**A**) or echocardiography (**B** and **C**). A negative correlation is observed between LVEF and increased asymmetry

Notably, higher mapping values did not correlate with reduced ventricular function in our study. However, an asymmetric myocardial involvement, particularly in ECV, displayed a significant negative correlation with LVEF (correlation coefficient (ρ)=-0.579, *p* = 0.004), as presented in Fig. 4. This correlation was absent with nT1 (ρ=-0.266, *p* = 0.220), T2 (ρ=-0.043, *p* = 0.945), and LGE (62 vs. 56.5, *p* = 0.235). The association persisted when categorizing patients based on the presence or absence of dysfunction, with the ECV asymmetry showing significance (4.6 vs. 2%, *p* = 0.003), while nT1 (54.5 vs. 22ms, *p* = 0.135), T2 (1.5 vs. 2ms, *p* = 0.473), and LGE (4/6 vs. 8/17, *p* = 0.365) remained unrelated.

Upon investigating heart function by echocardiography, and its association with CMR mappings, we consistently observed that the presence of LV dysfunction was linked to an asymmetric myocardial involvement, evident in nT1 (with a difference of 101.5 vs. 18 ms, *p* = 0.004) and ECV (with a difference of 6.4 vs. 2.2%, *p* = 0.012). LGE did not show statistical association (3/4 vs. 9/19, *p* = 0.329). A negative correlation (Fig. 4) was observed with LVEF and increased asymmetry, especially with nT1 (ρ=-0.472, *p* = 0.023) and ECV (ρ=-0.460, *p* = 0.049), but not with T2 (ρ=-0.381, *p* = 0.073). Additionally, asymmetrical nT1 mappings showed a negative correlation with superior s’ wave values in the lateral wall (ρ=-0.482, *p* = 0.02), reflecting LV function.

Finally, among dystrophinopathy patients, ECG alterations were associated with elevated LVPW mappings, particularly in nT1 (1087 vs. 1028 ms, *p* = 0.024), ECV (30.5 vs. 25.6%, *p* = 0.052, though not statistically significant). However, these alterations were not linked to lower LVEF (53.6 vs. 59%, *p* = 0.658), LV end-diastolic volume (63.5 vs. 75 mL/m^2^, *p* = 0.812), myocardial mass (73.5 vs. 62 g/m^2^, *p* = 0.87), or LGE (4/6 vs. 8/17, *p* = 0.640).

## Discussion

In this study, limited to pediatric patients, significant differences emerged between patients and controls in key cardiac parameters, including LVEF, myocardial mass, and LGE, confirming cardiac abnormalities in these patients. Tissue mappings, particularly nT1 and ECV, also displayed substantial variations, indicating their potential as sensitive indicators of myocardial involvement. Notably, higher mapping values did not correspond to reduced ventricular function. However, an asymmetric myocardial involvement, especially in nT1 and ECV, exhibited a significant negative correlation with LVEF, highlighting the nuanced relationship between tissue mappings and cardiac function in dystrophinopathies.

CMR with LGE stands as the gold standard for cardiac assessment in dystrophinopathies, assuming a fundamental role in risk stratification [1, 2, 6]. It offers a precise and reproducible method for evaluating LV volumes and function, along with the capability to detect fibrosis based on LGE, a feature strongly linked to adverse cardiac outcomes [9, 11, 18]. In our pediatric cohort, while their cardiac involvement is typically less severe than that reported in adult studies, we observed lower LVEF, increased myocardial mass, and the presence of LGE in comparison with controls, findings consistent with the existing literature [8, 10, 19]. LGE excels at identifying focal fibrosis, but its effectiveness is limited in the early detection of diffuse fibrosis, underestimating the extent of cardiac involvement [9, 12]. To enhance the early detection of cardiac affectation, it becomes imperative to evaluate subclinical microstructural fibrofatty infiltration, a task beyond the capabilities of LGE-CMR alone [8–10].

CMR parametric mappings provide valuable insights into the understanding of myocardial diseases in these patients [8, 10, 13]. In contrast to LGE imaging, mappings do not rely on the presence of normal myocardium for comparison [11]. In the context of dystrophinopathies, nT1 and ECV serve as estimates for diffuse myocardial fibrosis, although they can also be elevated in the presence of edema. Conversely, T2 values increase in response to fat (which is primarily characterized by low nT1 values) and edema, but are not indicative of fibrosis [12, 13, 20]. Our study revealed notable differences in myocardial nT1 and ECV measurements when compared to controls, aligning with existing literature [8, 10, 14, 19, 21]. However, we did not observe differences in T2-weighted mappings.

Current evidence suggests that, when CMR is performed, patients usually exhibit elevated myocardial nT1 and ECV levels, even with normal LVEF and absence of LGE, although higher mapping values often present together with LGE [8, 14, 19], This situation indicates the coexistence of severely damaged focal areas (LGE) and diffuse abnormalities in the remaining myocardium (mappings) [9]. The elevation of nT1 and ECV mappings is indicative of a diffuse expansion of the extracellular matrix, a well-known predictor of adverse outcomes, independent of the underlying disease [22, 23]. NT1 and ECV may serve as non-invasive biomarkers for early subclinical myocardial disease in this high-risk population [8, 19], offering the potential for monitoring disease progression, given their correlation with the degree of cardiovascular involvement [12, 14].

In our cohort, a myocardial involvement asymmetry was observed, which was significantly more pronounced in patients compared to controls. It is noteworthy that a greater degree of asymmetry was not associated with the presence of LGE. Interestingly, even healthy individuals have been reported to exhibit transient segmental T1 abnormalities, often indicative of transitory inflammation [24]. However, prior studies have consistently reported significantly increased nT1 and ECV values in the lateral wall, particularly in the inferolateral segment, while the septum appeared less affected [8, 10, 18, 19]. Although our study did not establish a direct link between asymmetric involvement and the presence of LGE, it is worth mentioning that LGE and elevated mappings often co-occur in the same region, reflecting the heightened mechanical stress experienced there. In this situation, LGE may correspond to a more severe damage when compared to the subtle and diffuse fibrosis that may only be detected by mappings in LGE-negative regions [8, 9].

We incorporated female carriers alongside BMD and DMD patients to comprehensively assess CMR findings across the spectrum of dystrophinopathies. While carriers typically experience milder cardiac involvement, they remain susceptible to cardiac dysfunction [5, 6, 21]. Interestingly we found and asymmetric involvement in nT1 values, with shorter nT1 values and prolonged T2 in our patient, when compared to controls. Although previous studies did not yield statistically significant mapping differences, a noticeable trend towards increased segmental ECV values was observed in the inferior and inferolateral myocardial walls [25].

The most striking finding in this study is that, while higher mapping values did not correlate with reduced ventricular function, an asymmetric involvement in nT1 and ECV demonstrated a significant negative association with LVEF. Patients with dystrophinopathies may exhibit elevated nT1 and ECV, even when their LVEF is within the normal range, revealing early cardiac involvement [9, 19]. However, no specific pattern of involvement has been directly linked to ventricular dysfunction thus far. A decreased LVEF has just been associated with more extensive cardiac damage, primarily indicated by LGE [8, 9], and more recently, by tissue mappings [10]. This discovery holds significant promise, especially in light of the lack of validated and sensitive cardiovascular imaging markers for early cardiac deterioration identification and monitoring treatment response, particularly in pediatric patients.

In our study, including pediatric patients with potentially less cardiac involvement compared to studies in the adult population, we did not find significant elevated T2 relaxation times, which is consistent with previous reports [19]. However, contrasting results have been documented, particularly in older patients with reduced LVEF, where elevated T2-mappings were noted in a number of patients; remarkably, those with elevated T2 also demonstrated increased nT1 and ECV in the same myocardial region [10]. In classical CMR, LGE cannot discriminate between edema, fibrosis, and fat, but parametric mappings, with the inclusion of T2, offer a comprehensive approach to tissue characteristics as T2 exhibit increases in response to fat and edema [9, 10]. Roughly, edema represents an initial response to cardiac damage, indicative of acute lesions irrespective of etiology [18, 20] while in dystrophinopathies it appears to play a pivotal role in the development of cardiac lesions, with a cyclical pattern of edema and fibrosis occurring until late-stage fatty deposition is detected [10]. Ideally, the combination of nT1, T2 and ECV could provide insights into the inflammatory, fatty and fibrotic components, but the relationship between these changes remains complex and nonlinear.

### Limitations

This study’s limitations include a small sample size, hindering extensive subgroup analyses and generalizability. Selection bias may also exist, as cardiac assessments began at age 10, potentially missing early cardiac involvement. The absence of endomyocardial biopsy data limits direct assessment of tissue changes, even though it is not routinely performed in these patients. The absence of longitudinal data limits insights into disease progression, and the impact of novel specific treatments on cardiac markers was not considered. Future studies with larger cohorts, broader age ranges, and longitudinal assessments, as well as potential inclusion of biopsy data, are needed for a comprehensive understanding of dystrophinopathy-related cardiac changes.

## Conclusions

In conclusion, CMR parametric mappings serve as a valuable tool for detecting microstructural damage in pediatric dystrophinopathies. Although mapping values may not directly relate to ventricular function, the observed negative correlation between asymmetric involvement in nT1 and ECV, and LV function, suggests its potential as an early indicator of myocardial dysfunction. Nevertheless, comprehensive research involving larger cohorts, a wider age range, and longitudinal data is imperative to gain a complete understanding of CMR changes and to enhance risk stratification.

## Data Availability

The data that support the findings will be made available from the corresponding author upon reasonable request.
